# How to Integrate Patient-Centered Measures in Routine Care: Lessons from Belgian Experiences

**DOI:** 10.1192/j.eurpsy.2022.68

**Published:** 2022-09-01

**Authors:** L. Jansen, F. Glowacz, A. Kinard, R. Bruffaerts

**Affiliations:** 1 KU Leuven, Public Health Psychiatry, Leuven, Belgium; 2 Université de Liège, Service De Psychologie Clinique De La Délinquance, Liège, Belgium

**Keywords:** Mental Disorders, Primary care psychology, Patient-reported outcome measures, Patient-reported experience measures

## Abstract

BACKGROUND Against the treatment gap and the long delays in seeking treatment for mental health problems, primary care psychology (PCP) was added to reimbursed outpatient mental health services in the Belgian healthcare system. PURPOSE Within the Evaluation of Primary Care Psychology study (EPCAP), which provides evaluation of the measure of reimbursement of PCP, the objectives were: (1) To describe the patient-reported outcome measures (PROMs) and patient-reported experience measures (PREMs) of patients treated with PCP and (2) formulate guidelines to integrate patient-related findings and experiences within community mental health services. METHOD 428 patients participated in an online survey at the start of their PCP treatment in Belgium and after 3 and 6 months. Besides sociodemographic characteristics, DSM-5 mental disorders, suicidality, and service use, they were questioned about their findings (PROMs) and experiences (PREMs). RESULTS Almost 90% met the criteria of a lifetime as a 12-month DSM-5 mental disorder or STB at the start of PCP treatment. Both subjective well-being and the proportion of patients who had positive experiences regarding their PCP treatment increased with 46% resp. 23.2% after 3 months and remained stable after 6 months. CONCLUSION Although PCP in Belgium serves a clinical patient population with high proportions of lifetime and 12-month mental disorders and suicidality, their subjective well-being increased after 3 months and remained stable after 6 months. Despite differences between groups of patients, PCP seems to have a positive effect on subjective well-being of these patient in short term. Integration of PROMs and PREMs into PCP were recommended.

**Disclosure:**

No significant relationships.

